# Gastrin-Releasing Peptide Signaling Plays a Limited and Subtle Role in Amygdala Physiology and Aversive Memory

**DOI:** 10.1371/journal.pone.0034963

**Published:** 2012-04-11

**Authors:** Frederique Chaperon, Markus Fendt, Peter H. Kelly, Kurt Lingenhoehl, Johannes Mosbacher, Hans-Rudolf Olpe, Peter Schmid, Christine Sturchler, Kevin H. McAllister, P. Herman van der Putten, Christine E. Gee

**Affiliations:** Novartis Institutes for Biomedical Research, Novartis AG, Basel, Switzerland; University of Toronto, Canada

## Abstract

Links between synaptic plasticity in the lateral amygdala (LA) and Pavlovian fear learning are well established. Neuropeptides including gastrin-releasing peptide (GRP) can modulate LA function. GRP increases inhibition in the LA and mice lacking the GRP receptor (GRPR KO) show more pronounced and persistent fear after single-trial associative learning. Here, we confirmed these initial findings and examined whether they extrapolate to more aspects of amygdala physiology and to other forms of aversive associative learning. GRP application in brain slices from wildtype but not GRPR KO mice increased spontaneous inhibitory activity in LA pyramidal neurons. In amygdala slices from GRPR KO mice, GRP did not increase inhibitory activity. In comparison to wildtype, short- but not long-term plasticity was increased in the cortico-lateral amygdala (LA) pathway of GRPR KO amygdala slices, whereas no changes were detected in the thalamo-LA pathway. In addition, GRPR KO mice showed enhanced fear evoked by single-trial conditioning and reduced spontaneous firing of neurons in the central nucleus of the amygdala (CeA). Altogether, these results are consistent with a potentially important modulatory role of GRP/GRPR signaling in the amygdala. However, administration of GRP or the GRPR antagonist (D-Phe^6^, Leu-NHEt^13^, des-Met^14^)-Bombesin (6–14) did not affect amygdala LTP in brain slices, nor did they affect the expression of conditioned fear following intra-amygdala administration. GRPR KO mice also failed to show differences in fear expression and extinction after multiple-trial fear conditioning, and there were no differences in conditioned taste aversion or gustatory neophobia. Collectively, our data indicate that GRP/GRPR signaling modulates amygdala physiology in a paradigm-specific fashion that likely is insufficient to generate therapeutic effects across amygdala-dependent disorders.

## Introduction

Pavlovian fear conditioning models associative fear learning, a process that is thought to be involved in the etiology of human anxiety [1]–[3]. The amygdala is a key neuroanatomical and physiological substrate for fear learning [4]–[6]. This structure relays information to autonomic and somatomotor centers that mediate specific fear responses [4], [7]. Fear conditioning induces long term potentiation (LTP)-like changes in thalamo- and cortico-amygdala synaptic transmission [8], [9] and both fear conditioning- and LTP-induced plasticity share common mechanisms of induction and expression (for review see [10], [11]).

Amygdala LTP and conditioned fear are under tight control of local inhibitory GABAergic interneurons. A wealth of clinical imaging data implicates hyperfunctioning of the amygdala in anxiety disorders such as social anxiety, phobias and post-traumatic stress disorder [12], [13] and there appear to be learning components in the etiology of these diseases [14], [15]. Neuropeptides may modulate anxiety- and stress-related behavioral effects through their actions on distinct subpopulations of neurons located in the lateral and/or central lateral (CeL) and central medial (CeM) amygdala nuclei. For example, the neuropeptide oxytocin which has strong anxiolytic effects, excites a subpopulation of CeM-projecting inhibitory neurons in the CeL [16]. Neuromodulatory projections that limit amygdala excitability likely serve to prevent the formation of exaggerated conditioned responses and pathological states such as anxiety (for review see [17]). Therefore, pharmacological agents that alter specific inhibitory activities in the amygdala or otherwise limit amygdala excitability may offer novel therapeutic strategies for the treatment of mood and anxiety disorders associated with amygdala hyperexcitability.

Gastrin-releasing peptide (GRP) is produced in the amygdala and excites local interneurons via the gastrin-releasing peptide receptor (GRPR). Mice deficient in GRPR show greater and more persistent fear memory after single-trial associative learning and it has been proposed that agonists may be developed as therapies for fear-related disorders [18]. GRPR also has a role in the regulation of immune function [19], itch [20] and is implicated in the pathogenesis of human cancers [21] which may limit the utility of activators as therapies.

To gain a better understanding of the specific versus more general role of GRP/GRPR signaling in the fear circuit, we assessed the role of GRP and its receptor in the amygdala, in single versus multiple-trial fear conditioning and in other amygdala-dependent paradigms.

## Results

### GRPR expression in the amygdala

**Figure 1:**
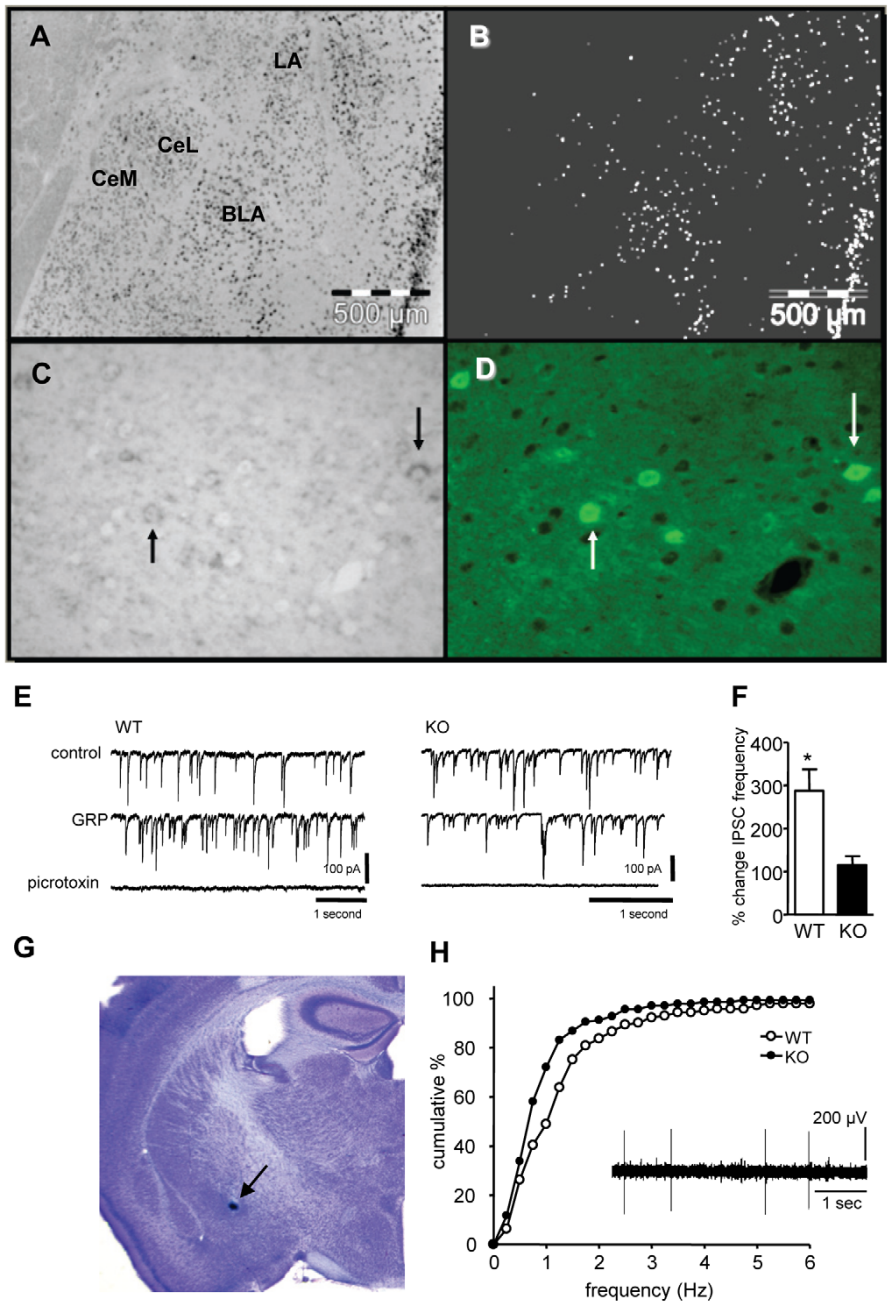
The gastrin-releasing peptide receptor is expressed in interneurons in the lateral amygdala and affects amygdala physiology. **A**) *In situ* hybridization of the GRPR in the amygdala. **B**) Binary version of A) that more clearly distinguishes the ISH signal (white dots), mainly in the lateral (LA) and basolateral (BLA) with only very few labeled cells in the central lateral (CeL) and central medial (CeM) nuclei. **C**) Higher power image showing ISH signal in neurons that were **D**) co-immunolabeled for eGFP being expressed under control of the GAD67 promoter. **E**) Sample recordings of spontaneous inhibitory postsynaptic currents recorded from LA pyramidal neurons in control conditions, in the presence of GRP and after addition of picrotoxin in slices made from WT and GRPR KO mice. The patch pipette contained high Cl^−^ therefore IPSCs were inward at the holding potential of −70 mV. CNQX (20 µM) was present to block fast excitatory activity. Picrotoxin (100 µM) blocked all the inward currents confirming their inhibitory nature. **F**) Quantification of the results from 6 slices from WT and 4 slices from GRPR KO mice. **G**) Typical example of the most rostral and ventral *in vivo* recording position in the central medial nucleus of the amygdala. **H**) Cumulative frequency plot of CeM single unit activity from 12 WT and 12 GRPR KO mice. Inset shows a sample record.

To determine which cell types in the mouse amygdala express GRPR we used combined i*n situ* detection of GRPR mRNA and immunofluorescent detection of eGFP in GAD67-eGFP mouse brain sections. GRPR mRNA was mostly co-localized with eGFP in a subset of GAD67-eGFP neurons (Fig. 1 C, D). In the LA and basolateral amygdala (BLA) GRPR mRNA was expressed primarily in GAD67-eGFP positive GABAergic neurons (Fig. 1A, B). GABAergic neurons in the intercalated cell masses lacked GRPR mRNA. In the central amygdala (CeA), the lateral nucleus (CeL) contained a dispersed set of GAD67-eGFP neurons expressing GRPR but the medial nucleus (CeM) was largely devoid of such cells.

#### GRP increases inhibitory activity in lateral amygdala principal neurons

Administration of GRP to amygdala slices *in vitro* enhanced the number of spontaneous inhibitory currents recorded from principal neurons in the LA. Addition of 200 nM GRP increased spontaneous IPSCs in slices from wild-type (WT, paired *t*-test; p = 0.046, n = 6) but not GRPR knock-out mice (GRPR KO, paired *t*-test; p = 0.42, n = 4; Fig. 1E, F). The control IPSC frequencies were not different between the genotypes (WT 4.3±0.9 s^−1^, n = 6; KO 8.3±2.8 s^−1^ n = 4, p = 0.14). Picrotoxin blocked all inward currents confirming that these were mediated by activation of fast GABA_A_ receptors (Fig. 1E). Activation of GRPR by GRP therefore increased spontaneous inhibitory activity in LA pyramidal neurons in agreement with earlier findings [18]. Since the LA is thought to be the principle site where conditioned-stimulus (CS)-unconditioned stimulus (US) associations are formed, GRP/GRPR signaling in the LA is likely at least part of the mechanism via which this cascade influences the acquisition and/or expression of associative fear memory.

### Reduction of single-unit firing frequencies in CeM neurons in vivo

The CeA is the principle output structure of the amygdaloid complex. Output neurons that mediate endocrine, autonomic and motor aspects of fear responses are mainly located in CeM, which in turn is under inhibitory control from the CeL. Fear conditioning leads to increased activity of LA neurons, which can project to CeL [22] and to decreased basal firing of CeM neurons [23]. Since GRPR expressing neurons are located both in LA and CeL, we tested whether GRPR ablation changed baseline activity in CeM. Single unit recordings were conducted from the CeM of anaesthetized mice (example of most rostral and ventral recording site Fig. 1G). The results show that the majority of neurons in CeM fired at frequencies below 3 Hz. A total of 141 single units were recorded from 12 WT mice and 136 single units were recorded from 12 GRPR KO mice. The mean firing rate of CeM neurons was 1.32±0.12 s^−1^ in the WT and 0.91±0.09 s^−1^ in the KO mice. Kolmogorov-Smirnov analysis indicated that the distribution of firing frequencies of CeM neurons were different in the WT and GRPR KO mice (p<0.001; Fig. 1H). These findings show that GRPR ablation decreases basal firing rates of CeM neurons.

### Enhanced fear responses in GRPR KO mice following single-trial conditioning

**Figure 2:**
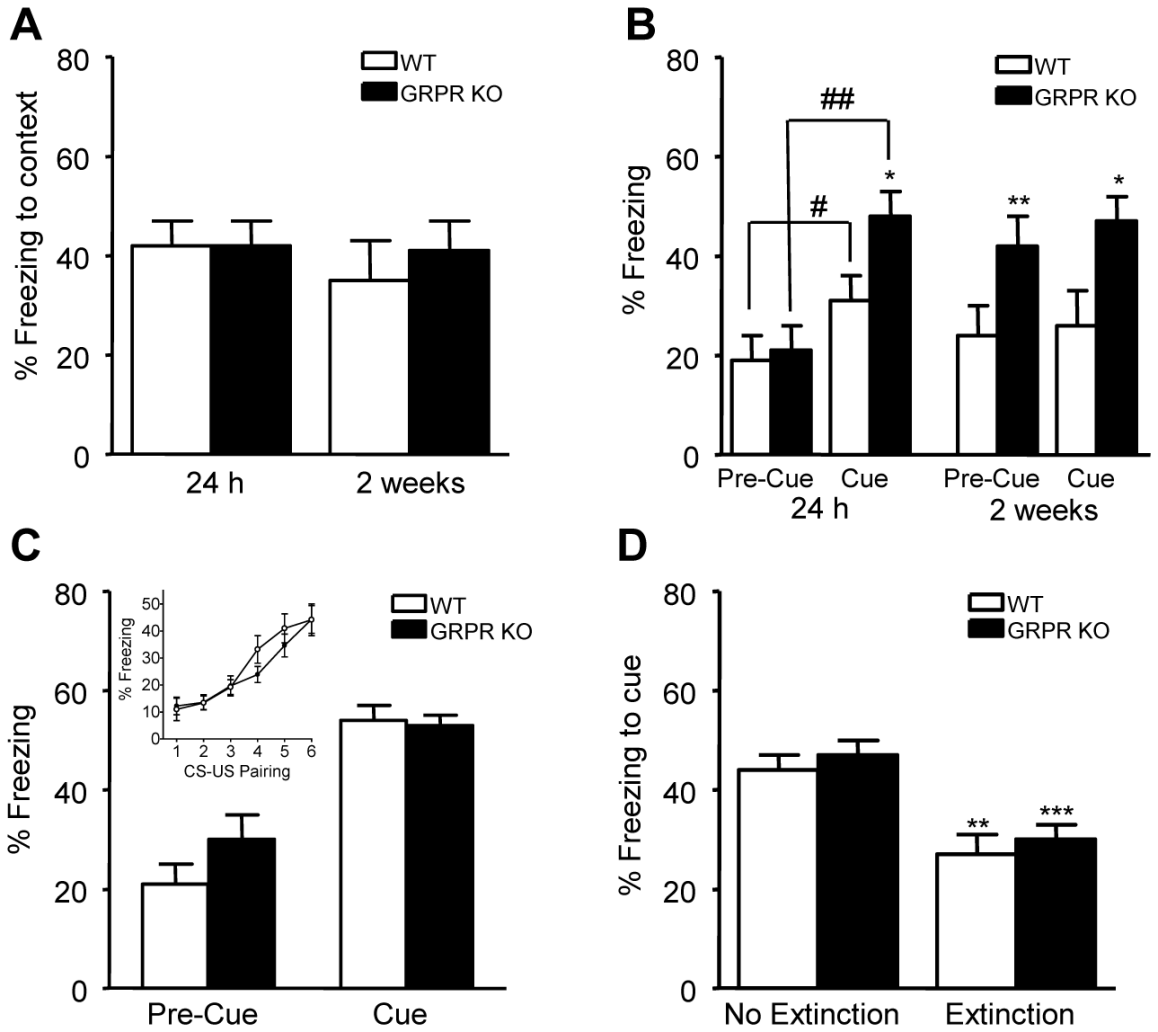
Expression of conditioned fear is altered in GRPR KO mice after single-pairing but not multiple-pairing conditioning. **A,B**) Fear conditioning was induced by a single CS-US (tone-shock) pairing in context 1. 24 h and 2 weeks later the freezing response in the same context was tested **A** and response to the cue alone was tested in a new context **B** (WT n = 12, GRPR KO n = 11). **C,D**) To test for extinction of conditioned fear GRPR KO (n = 12) and WT mice (n = 11) were subjected to multiple CS-US (tone-shock) pairing in context 1. Freezing levels during acquisition are shown in the inset in **C**. **C**) At the start of extinction training, baseline (pre-cue) and cue-related freezing responses were tested in a new context. **D**) At the end of 4 days of extinction training, the freezing response to the cue was tested in mice that were handled but not given the training (no Extinction; n = 12) and mice subjected to extinction training (Extinction; 10 presentations of CS alone each day).

To evaluate whether the decreased firing rates of CeM neurons in GRPR KO mice translate to changes in amygdala-dependent behavior, we tested GRPR KO mice in a fear conditioning paradigm. Using a one trial fear conditioning protocol, we found that during the single pairing of a tone with a foot shock, the levels of freezing were not significantly different in GRPR-deficient mice and WT littermates (*t*-test: p = 0.33) (data not shown). In addition, the reactivity to the aversive stimulus (electric foot shock) was similar in mice of both genotypes (movement velocity: mean ± sem in cm/s: WT 52±3, KO 54±2, *t*-test p = 0.52). When the mice were re-exposed to the conditioning context 24 h after the training, both mutant and WT animals exhibited 42% contextual freezing during the 3 min retention test (Fig. 2A). In the re-test performed 2 weeks later, this response was not modified and no difference in freezing due to genotype was observed (two-way ANOVA: Genotype: F_(1,21)_ = 0.18; p = 0.67; Time: F_(1,21)_ = 0.83; p = 0.37; Genotype×Time: F_(1,21)_ = 0.55; p = 0.46). Three hours after each contextual retention test, the mice were placed in a novel environment and submitted to a retention test for the cue (Fig. 2B). When the animals were tested 24 h after conditioning, all displayed an increase in freezing during the tone presentation (Cue) as compared to the freezing prior to the tone (Pre-Cue) (two-way ANOVA: Genotype: F_(1,21)_ = 2.50; p = 0.13; Test condition: F_(1,21)_ = 24.99; p<0.001; Genotype×Test condition: F_(1,21)_ = 2.65; p = 0.12; post-hoc paired *t*-tests WT: p = 0.023, GRPR KO: p = 0.002). However, as shown in Fig. 2B GRPR KO mice froze significantly more than the WT mice (ANOVA: Genotype: F_(1,21)_ = 5.27, p = 0.03), demonstrating that 24 h after single trial fear-conditioning, the absence of GRPR enhanced the expression of learned fear as reported by Shumyatsky et al. [18]. When the mice were retested 2 weeks after the conditioning, GRPR KO as compared to WT mice showed significantly larger freezing responses both during the pre-cue period (ANOVA: Genotype: F_(1,21)_ = 7.81, p = 0.01) and during the cue presentation (ANOVA: Genotype: F_(1,21)_ = 4.57, p = 0.04; Fig. 2B). It is important to note, however, that freezing did not increase when the cue was presented but remained the same as in the pre-cue period (two-way ANOVA: Genotype: F_(1,21)_ = 7.59, p = 0.01 Trial condition: F_(1,21)_ = 0.04; p = 0.84; Genotype×trial condition: F_(1,21)_ = 0.88, p = 0.36). Thus, whereas 24 h after single-trial conditioning the GRPR KO mice showed an enhanced fear response to the conditioned cue, 2 weeks later they showed a generalized enhanced freezing response that was unspecific to the cue.

### Lack of GRPR does not affect multiple-trial fear learning and extinction

The previous experiment used a simple and fairly weak single-trial protocol to induce associative fear learning. We went on to examine whether the learning induced by multiple CS-US pairings would also be modified by the lack of GRPR. When the CS and US were paired 6 times, freezing during the tone increased with the repeated tone-shock pairings (Fig. 2C, inset, two-way ANOVA: Trial number: F_(5,225)_ = 24.09; p<0.001). There was, however, no significant difference between WT and GRPR KO mice during the conditioning (Genotype: F_(1,45)_ = 0.54; p = 0.47; Genotype×Trial number: F_(5,225)_ = 0.66; p = 0.65). The reactivity to the foot shock (velocity) was similar in both genotypes (WT: 47.5±1.4 cm/s; KO: 48±1.2 cm/s, ANOVA: Genotype: F_(1,45)_ = 0.07; NS). Twenty-four hours after conditioning, the freezing response induced by the cue was evaluated in all mice prior to the extinction procedure (Fig. 2C). Although freezing during the pre-cue period was slightly higher in GRPR KO mice, this was not significantly different (ANOVA: Genotype: F_(1,45)_ = 1.84, p = 0.18). Likewise, there was no significant difference between WT and GRPR KO mice in the expression of cue-induced freezing (respectively, 54 and 53% of cue-induced freezing) (ANOVA Genotype: F_(1,45)_ = 0.11; p = 0.74). After completing the 3 days of extinction training, mice of both genotypes displayed significantly less freezing in response to cue presentation (p<0.001) than did their respective ‘No Extinction’ group during the final retention test on day 5 (Fig. 2D). A two-way ANOVA indicated that there was a significant effect of the extinction procedure, but no effect of genotype and no interaction between both factors (Extinction group: F_(1,43)_ = 25.06; p<0.001; Genotype: F_(1,43)_ = 0.96; p = 0.33; Extinction group×Genotype: F_(1,43)_ = 0.004; p = 0.95). Thus, when a stronger multiple CS-US pairing fear-conditioning protocol is used, there is no longer a significant effect of GRPR deletion on the conditioned fear response.

### Absence of GRPR alters short but not long-term plasticity in amygdala slices

**Figure 3:**
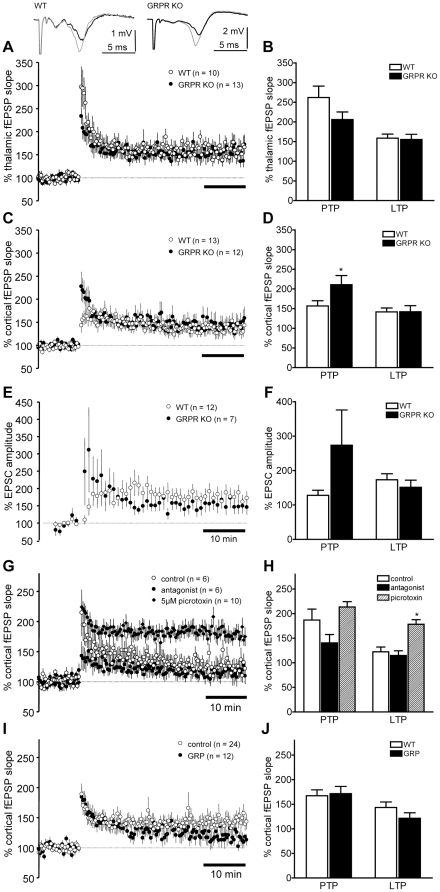
Long-term potentiation in the LA is not changed in GRPR KO mice or by agonist/antagonist application. **A**) Thalamic afferents were stimulated to evoke field excitatory postsynaptic potentials (fEPSPs) in the LA. Inset shows sample averaged traces (10 sweeps) from the 10 min baseline period (black) immediately before applying the tetanus (5×100 Hz/1 s trains, 20 s inter-train interval) and 40 min after the tetanus (grey). **B**) Mean ± s.e.m. of the change in fEPSP slope in the first 2 minutes after the tetanus (PTP) and 30–40 min after the tetanus. **C,D**) As in A,B except that cortical afferents were stimulated to evoke fEPSPs in the LA. **E,F**) Cortical afferents were stimulated at 30 s intervals to evoke EPSCs recorded from LA pyramidal neurons at −70 mV with the whole-cell voltage clamp technique. After a 10 min baseline 80 stimuli at 2 Hz were paired with depolarization to 30 mV. **G,H**) Long-term potentiation of cortico-LA fEPSPs induced by 5×100 Hz/1 s trains was not affected by bath application of 1 µM (D-Phe^6^,Leu-NHEt^13^,des-Met^14^)-Bombesin(6–14). Reducing inhibitory inputs by addition of 5 µM picrotoxin increased LTP. **I,J**) 1 µM GRP also did not significantly affect cortico-LA LTP.

We examined whether synaptic plasticity was altered in amygdala slices of GRPR KO mice. Field potentials (fEPSPs) in the LA, evoked by stimulation of thalamic inputs, were significantly potentiated following 5 trains of 100 Hz/1 s stimulation in both GRPR KO and WT littermates (Fig. 3A, B). In the first 2 min following the tetanic stimulation, the amount of post-tetanic potentiation of thalamo-LA synapses was not significantly different between acute slices from WT and GRPR KO mice (*t*-test, p = 0.13, Fig. 3A, B). There was also no significant difference in the amount of long-term potentiation (LTP), measured 30–40 min post-tetanus, between slices from GRPR KO and WT littermates (*t*-test, p = 0.85; Fig. 3A, B). Similarly, there was no difference in cortico-LA LTP between GRPR KO and WT mice (*t*-test, p = 0.35; Fig. 3C, D). However, PTP of the cortico-LA fEPSP slope was larger in amygdala slices from GRPR KO mice (*t*-test, p = 0.05; Fig. 3C, D). These findings suggest that the absence of GRPR in the LA mainly affected short-lasting synaptic plasticity.

We next tested whether pairing of postsynaptic depolarization of whole-cell patch-clamped pyramidal neurons in the LA with presynaptic stimulation of the cortical inputs at 2 Hz, would better reveal differences in cortico-LA plasticity in GRPR KO mice. As LTP is highly susceptible to the washout of postsynaptic second messengers, we applied the pairing paradigm within 10 min of gaining whole-cell access and restricted our comparisons to only those experiments in which there was significant LTP. There was no significant difference in the amount of LTP in GRPR KO and WT mice (*t*-test, p = 0.44; Fig. 3E, F). The amount of PTP again tended to be higher in the recordings from the GRPR KO mice (*t*-test, p = 0.08; Fig. 3E, F). Thus, in our hands genetic deletion of GRPR failed to affect thalamic-LA LTP and cortico-LA LTP, irrespective of its mode of induction using either trains of tetanic stimuli or pairing of postsynaptic depolarization with presynaptic stimulation.

### GRP and GRPR antagonists do not affect cortico-LA LTP in amygdala slices

In amygdala slices of WT mice, the GRPR antagonist (D-Phe^6^, Leu-NHEt^13^, des-Met^14^)-Bombesin (6–14) (1 µM) had no effect on LTP of cortico-LA fEPSPs (*t*-test, p = 0.59; Fig. 3G, H). Blocking of GRPRs would be expected to reduce the activation of interneurons during the induction of LTP. To ensure that our experimental conditions permitted detection of an enhancement of LTP by such a mechanism, we added a low concentration of picrotoxin (5 µM) to partially block inhibition. Reducing inhibition significantly increased the amount of LTP induced by tetanic stimulation (*t*-test vs control, p = 0.001; Fig. 3G, H) suggesting that our experimental conditions were not confounded by a ceiling effect that would have prevented detection of GRPR antagonist effects on LTP. Application of 200 nM GRP also did not significantly reduce cortico-LA LTP (Fig. 3I, J; *t*-test, p = 0.22). Thus, consistent with the multiple-trial conditioning protocols and the effects of genetic deletion of GRPR, we found no significant effect of GRP or the antagonist on cortico-LA synaptic plasticity.

### Intra-amygdala administration of GRP or a GRPR antagonist do not affect conditioned freezing

**Figure 4:**
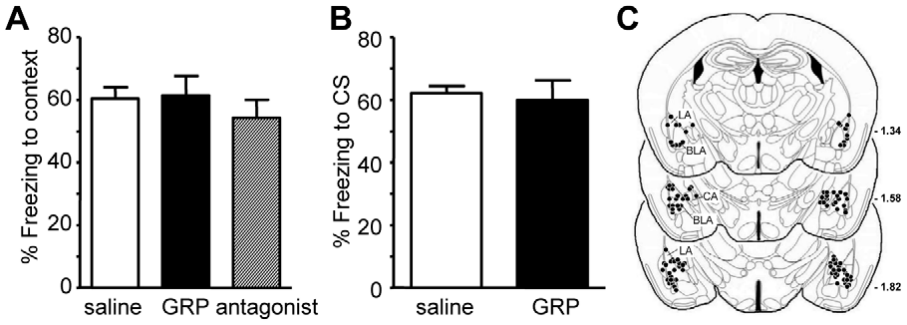
Exogenous GRP or GRPR antagonist did not affect expression of conditioned fear. **A**) 600 ng GRP or 3000 ng GRPR antagonist (D-Phe^6^,Leu-NHEt^13^,des-Met^14^)-Bombesin(6–14) was infused into the amygdala of C57BL/6 mice, that were conditioned with 6 CS-US pairings as in Fig. 2C, 10 min prior to testing freezing in the conditioning context 24 h later. **B**) Effect of intra-amygdala infusion of 600 ng GRP 10 min prior to testing freezing in response to the CS. **C**) Location of the bilateral injection sites determined from post-hoc histological analysis.

Genetic deletion of important receptor systems may induce compensatory mechanisms. Therefore, we tested whether acute bilateral intra-amygdala administration of GRP or the GRPR antagonist (D-Phe^6^, Leu-NHEt^13^, des-Met^14^)-Bombesin (6–14) affected the expression of learned fear in C57BL/6 mice. Of the 75 animals used for the experiments 17 animals (22%) had to be excluded from the final analysis after inspection of the injection sites because of either misplaced injections or lesions of the amygdala (Fig. 4C shows the injection sites). All the mice used in this experiment were first conditioned with the identical multiple-trial fear conditioning protocol as described above when comparing GRPR WT and KO mice. During conditioning, freezing increased significantly (ANOVA, Trial type: F's>11.05, p's<0.001) but no differences between the groups were observed (ANOVA, Group and interaction Group×Trial type: F's<1.23, p>0.28; data not shown). One day after conditioning, GRP or the GRPR antagonist were injected 10 min prior to the retention test for either context-induced freezing or, in a separate group of animals, for cue-induced freezing. Intra-amygdala infusions of GRP or (D-Phe^6^, Leu-NHEt^13^, des-Met^14^)-Bombesin (6–14) had no statistically significant effect on context-induced freezing (Fig. 4A, ANOVA Treatment: F_(2,32)_ = 0.49, p = 0.62). Likewise, infusion of GRP 10 min before the retention test had also no significant effect on cue-induced freezing (Treatment: F_(1,21)_ = 0.12, p = 0.74, Fig. 4B). As effects of intra-amygdala GRP and a GRPR antagonist were shown to be restricted to context-induced freezing in rats, we decided to spare animals and not to test the antagonist effects on cued fear [24], [25]. Altogether, intra-amygdala administration of GRP or a GRPR antagonist showed no significant effects on fear responses after multiple-trial fear conditioning.

### Lack of GRPR does not affect conditioned taste aversion (CTA)

**Figure 5:**
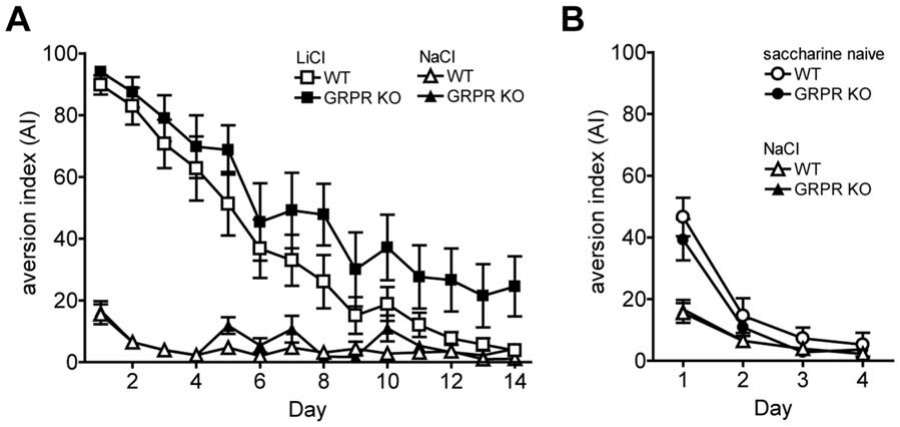
GRPR KO animals showed no differences in conditioned tast aversion (CTA) or neophobia. **A**) CTA was evoked by pairing a novel taste, saccharin, with a LiCl injection to induce illness the day before testing (LiCl; n = 12 mice per group). Control animals were offered the novel taste saccharin but injected with NaCl (NaCl groups; n = 11 mice per group) or given only water to drink and injected with NaCl the previous day (saccharin naive groups; n = 12 mice per group). **B**) Attenuation of neophobia and neophobia were assessed by comparing the aversion to saccharin on first exposure (saccharin naive) with the aversion shown by mice that were exposed to saccharin the previous day (NaCl). On successive days the neophobia was attenuated by repeatedly being given the chance to drink saccharin flavored water.

To test whether other forms of aversive memory might be sensitive to GRP/GRPR signaling, we tested whether GRPR ablation influenced conditioned taste aversion (CTA) and/or gustatory neophobia. On the day of conditioning, mice of both genotypes readily consumed saccharin solution and were then injected with either LiCl or NaCl solution (saccharin solution intake; WT: 1.70±0.09 ml LiCl injected group; 1.81±0.06 ml NaCl injected group; GRPR KO: 1.96±0.08 ml LiCl injected group; 1.81±0.07 ml NaCl injected group; F_(1,42)_ = 2.73; p>0.1). A third group of WT and KO mice did not receive saccharin and were given only water to drink prior to injecting NaCl. Both conditioned WT and GRPR KO mice (LiCl-treated animals) developed similar, robust levels of CTA to saccharin and preferentially drank water on day 1 after the conditioning (Fig. 5A). When compared to the saccharin-exposed animals that received NaCl injections, two factor ANOVA (factors: genotype, treatment) indicated that there was no significant difference in aversion index (AI) on day 1 between WT and GRPR KO mice (F_(1,42)_ = 0.95; p>0.3), no genotype×treatment interaction (F_(1,42)_ = 0.31; p>0.5), but a highly significant difference in AI between the groups that received LiCl versus NaCl injections (F_(1,42)_ = 739.6; p<0.001, Fig. 5A). When compared with the group that never received saccharin, there was no difference in AI on day 1 between WT and GRPR KO mice (F_(1,43)_ = 0.10; p>0.7), no genotype×treatment interaction (F_(1,43)_ = 1.46; p>0.2), but a highly significant effect of LiCl (F_(1,43)_ = 103.9; p<0.001). In summary, both WT and GRPR KO mice developed an equally robust CTA to saccharin when its first exposure was paired with LiCl-induced sickness. When offered the choice of drinking saccharin or water on each of the next 14 days, all LiCl-treated animals showed extinction of the CTA irrespective of genotype (Fig. 5A) and repeated-factor ANOVA (factors: genotype, day as repeated factor) revealed no significant difference between WT and GRPR KO mice (F_(1,22)_ = 2.12; p = 0.16), no significant genotype×day interaction (F_(13,286)_ = 0.65; p = 0.61) but a highly significant effect of day (F_(13,286)_ = 53.6; p<0.001, Fig. 5A). Altogether, these findings suggest that GRPR signaling does not play a significant role in the acquisition, expression and extinction of CTA.

The expression of neophobia to novel tastes in rodents is highly dependent on amygdala function and is intricately involved in the expression of CTA. We therefore also assessed whether WT and GRPR KO animals either expressed different levels of neophobia or showed differences in its attenuation. On day 1, animals naive to saccharin had a significantly higher AI than animals that were given saccharin the previous day (NaCl group). This behavior typically reflects mice exhibiting neophobia to saccharin on first exposure (F_(1,41)_ = 27.1; p<0.001, Fig. 5B). There was however, no difference in AI between WT and GRPR KO mice (F_(1,41)_ = 0.36; p>0.5) and no conditioning group×genotype interaction (F_(1,41)_ = 0.69; p>0.4). Attenuation of neophobia was subsequently achieved by a repeated several-day exposure of the mice to saccharin (without LiCl-induced malaise). As a result, all mice independent of genotype, drank almost exclusively saccharin when given the choice (Fig. 5B). These findings suggest that GRPR KO mice and their WT littermates show similar levels of innate fear and its attenuation as measured by gustatory neophobia.

## Discussion

Our data showed that GRP/GRPR signaling in the amygdala increased inhibitory activity in the LA, modulated single-unit firing frequency in the CeM nucleus and altered short- but not long-term synaptic plasticity in the LA. These physiological changes in the amygdala might explain enhanced fear responses in GRPR KO mice following single-trial conditioning but they are insufficient to significantly affect multiple-trial fear learning and extinction or other forms of associative aversive memory such as CTA. We saw that short-term plasticity in the cortico-LA pathway was enhanced in GRPR KO mice, suggesting that GRP/GRPR signaling limits the activation of this pathway in WT mice. We confirmed that GRPR is localized in a subset of GABAergic interneurons in the amygdala and that GRP application increased spontaneous IPSC frequency in LA pyramidal neurons suggesting that GRP indeed stimulates GABAergic interneurons in the LA [18], [26]. The LA is thought to be the principle site where CS-US associations are formed (for review see [22]). Therefore, GRP/GRPR signaling in the LA likely accounts for at least part of the mechanism via which the neuropeptide GRP influences the acquisition and/or expression of associative fear memory for weak single-trial conditioned stimuli. Importantly, genetic and pharmacological manipulation of GRP/GRPR signaling did not affect experimentally evoked cortico- and thalamic-LA LTP, nor did it affect the formation and expression of strong multiple CS-US pairing-evoked conditioned fear memories. The evidence that inhibition in the amygdala plays an important role in fear learning and extinction, is overwhelming [23], [27]. Interestingly, our results suggest that the activity of subclasses of inhibitory neurons other than or in addition to those sensitive to GRP would have to be targeted to interfere with strongly conditioned responses [28]–[30]. Furthermore, modulation via GRP/GRPR signaling is not apparent for all learned aversive responses including CTA, which is largely independent of motor activity and considered a rather mild form of aversive learning [31], [32]. Finally, GRPR ablation also did not affect gustatory neophobia, a kind of innate fear against novel tastes that is highly amygdala-dependent and required for CTA [33].

We observed that GRPR KO mice showed enhanced freezing response to cue 24 h after single-trial fear conditioning, but we did not confirm the effects on contextual freezing or the persistence of fear memory reported by Shumyatsky et al., [18]. Similar CS-US parameters produced lower freezing responses in our hands (<60% vs about 80%) and this might provide one explanation why fear memory in GRPR KO mice was less persistent under our experimental conditions. The lack of effect on LTP in our study is also in contrast to the earlier finding that LTP is enhanced in amygdala slices from mice lacking GRPR [18]. We attempted to precisely replicate the experimental design of the earlier report. Non-identical experimental housing conditions and/or breeding history might have contributed to these differences [34]. Mice lacking for example the closely related bombesin receptor 3 show alterations in weight gain, stereotypic movement and social responses when housed singly vs in groups [35]. It has been reported that chronic corticosterone exposure potentiates stressor-elicited GRP release in the CeA. Therefore, GRP/GRPR signaling might be particularly sensitive to stress levels [36]. It is also noteworthy that others are unable to exactly replicate the originally reported phenotype of enhanced fear to both context and cue in the GRPR KO mice [37].

To try and rule out confounds such as compensatory changes in GRPR KO mice we examined *in vivo* and *in vitro* the effects of exogenous GRP and a GRPR antagonist. Numerous studies have highlighted the role of amygdala LTP as a physiological correlate of fear learning (reviewed in [38], [39]. In the present study, LA LTP was unchanged after application of either exogenous GRP or a GRPR antagonist. Furthermore, when injected into the amygdala *in vivo*, these compounds also failed to modify the expression of context and cue conditioned fear after multiple trial conditioning. Earlier reports documented that GRP injected into the rat CeA, prelimbic and infralimbic cortices reduced conditioned freezing [40]. Central (i.c.v) administration of GRP has also been reported to reduce fear-potentiated startle and conditioned freezing responses. The GRPR antagonist RC-3095 was shown to block the reduction of context and cued fear normally observed over time [41] also see [42]. On the contrary, infusion of either GRP or the GRPR antagonist RC-3095 into the BLA has anxiolytic-like effects on the expression of conditioned freezing [25]. Roesler et al., [43] showed that systemic or intra-amygdala (BLA) injection of RC-3095 impaired aversive memory consolidation without altering object recognition memory. Thus, anxiolytic-like effects have been reported for both activation and blockade of GRPR-mediated signaling suggesting that it is exceedingly difficult to predict the overall effect of systemic brain-penetrating GRPR agonists/antagonists. Indeed, systemic application of GRP or its amphibian homologue bombesin enhances memory retention following aversive training protocols. This effect is attenuated by vagotomy and transient inactivation of the NTS or amygdala suggesting that a systemic agonist may have opposing effects at the periphery and in the CNS and may enhance rather than decrease fear [44], [45].

In conclusion, our results collectively with earlier reports indicate that GRP/GRPR signaling plays a subtle and complex role in amygdala physiology. Increasing inhibitory activity in the amygdala via activation of GRPR clearly modulates amygdala physiology and some paradigm-specific forms of emotional memory. However, these effects are not potent enough to significantly attenuate strongly conditioned aversive learning experiences making it unlikely that GRPR modulation would be a broadly applicable therapeutic principle for amygdala-dependent disorders.

## Materials and Methods

### Ethics Statement

All experiments were conducted in accordance with international guidelines and the Swiss Law for the care and use of animals and were approved by the Kantonales Veterinäramt Basel-Stadt.

### Animals

Male GRP receptor knock-out mice (backcrossed N>9 generations to the C57BL/6J strain, [46], were bred and raised in the Novartis SPF-breeding facility. Genotyping was performed by RT-PCR. C57BL/6J mice were purchased from Janvier (France) or Charles River (Germany). One to 5 mice were housed in each cage. Food (Provimi Kliba SA; Kaiseraugst, Switzerland) and water were available *ad libitum*. Mice 2–3 months old were used for all behavioral and electrophysiology experiments except for the patch-clamp recordings, which were performed in brain slices prepared from 4–6 week old mice.

### Histology

#### In situ hybridization

20 µm coronal sections were prepared from fresh frozen mouse (10 to 12 week old male C57BL/6) brains, fixed for 1 h in PBS buffered 4% paraformaldehyde, dehydrated in increasing ethanol solutions and subjected to an automated ISH procedure (VENTANA Discovery XT technology). Briefly, sections were postfixed for 4 min with VENTANA RiboPreb™ solution and conditioned by heat denaturation (12 min at 98°C in citrate buffer, pH 6.0) followed by mild protease treatment (incubation with VENTANA protease III for 4 min at 37°C). Sections were then hybridized for 6 h at 65°C with 1 ng/µl digoxigenin-labeled antisense RNA (corresponding to nucleotides 638–1092 of mouse cDNA), diluted in hybridization solution containing one part VENTANA RiboHybe™, and two parts 2×SSC, followed by high stringency washing with 2×SSC at 75°C for 3×8 min, and post-fixation for 8 min in VENTANA RiboFix™. To visualize hybridization signals, sections were incubated for 28 min with alkaline phosphatase labeled sheep anti-digoxygenin Fab fragments (Roche Diagnostics) diluted 1∶500 in VENTANA discovery antibody diluent, and subjected for 9 h to an alkaline phosphatase-catalized color reaction with NBT/BCIP (VENTANA BlueMap kit).

#### Dual in situ/immunofluorescence labeling

For dual *in situ* and immunofluorescence staining to visualize GAD67 expressing GABAergic interneurons, 4 µm coronal sections from paraffin embedded GAD67-eGFP transgenic mouse brains were subjected to an automated ISH procedure followed by immunofluorescence staining of eGFP. Briefly, paraffin sections were de-waxed, postfixed and conditioned by heat pre-treatment and moderate proteolysis (VENTANA protease III for 8 min at 37°C). Hybridization, washing and digoxygenin immunostaining was performed as described. For eGFP immunofluorescence staining, sections were incubated for 2 h at room temperature with a goat anti-eGFP antibody (Abcam; diluted 1∶200) followed by incubation for 1 h at room temperature with ALEXA 488 labeled donkey anti-goat IgG (INVITROGEN). Slides were analyzed by dual brightfield and fluorescence microscopy (Olympus BX51) and digital imaging (ColorView II camera and AnalySIS software, Soft Imaging Systems).

#### In vivo electrophysiology

Mice were anaesthetized with 3.6 g/kg intraperitoneal urethane. The animals were fixed in a stereotaxic holder and body temperature was maintained around 37.2°C with a heating pad. Animals received 1 ml of isotonic saline solution intraperitoneally every hour. The skull was exposed with one longitudinal midline cut and a hole for the recording electrode was made 1 mm caudal to Bregma and 2.6 mm lateral from the midline. To record CeA neuron activity, a glass electrode filled with 2 M NaCl and 2.5% pontamine sky blue (tip broken to about 3 µm) was introduced through the opening. In each animal, 4 recording electrode tracks were made in a parasagittal plane (0.8, 0.9, 1.0, and 1.1 mm caudal to Bregma, 2.6 mm lateral from the midline). Recordings were made from all neurons encountered between 3.8 and 4.5 mm below the cortical surface. Extracellular single units were AC coupled and amplified with a Grass P16 amplifier and acquired using custom-written software running in LabView (New Visions Engineering, Switzerland). Single units were distinguished using a window discriminator and average spontaneous firing frequency was calculated from 50 3 s sweeps collected every 6 s. For histological verification of the recording sites, pontamine sky blue was ejected from the recording pipette at the last recording site. Brains were then processed for histology to verify recording sites as described below.

#### In vitro electrophysiology

Mice were anaesthetized with isoflurane and killed by decapitation. Brains were rapidly removed and coronal slices (350–400 µM thick) containing the amygdaloid complex were cut with a Leica VT vibratome or a Microm vibratome in ice-cold saline equilibrated with 95%O_2_/5%CO_2_ containing (in mM): NaCl 124; KCl 2.5; KH_2_PO_4_ 1.2; CaCl_2_ 2.5; MgSO_4_ 1.3; NaHCO_3_ 26, glucose 10, saccharose 4 (pH 7.4, osmolarity adjusted to 320±2 mOsm by reducing amount of H_2_O). After cutting slices were maintained in the same solution but fully diluted to give osmolarity 306±2 mOsm at room temperature.

For field recordings, slices were transferred to an interface-type recording chamber and superfused with the above solution at 27°C. Stimulation and recording electrodes were positioned to activate either cortical or thalamic inputs to the LA [47]. Stimuli were delivered with a constant current stimulus isolation unit to evoke a fEPSP that was 25–40% of the maximum. Responses were recorded with an Axoprobe 1A amplifier and pClamp 9.0 software. Data were analyzed with custom written analysis routines in VBA and Excel. After recording test responses at 30 s intervals to obtain a 10 min baseline period, LTP was induced with 5×1 s trains of 100 Hz stimuli at the test amplitude delivered every 20 seconds. Data were normalized to the baseline fEPSP slope and are expressed as mean ± SEM.

For whole-cell patch clamp recordings slices were superfused in a submersion chamber with ACSF containing: (in mM) 119 NaCl, 2.5 KCl, 2.5 CaCl_2_, 1.0 MgSO_4_, 1.25 NaH_2_PO4, 26.0 NaHCO_3_, 10 glucose, equilibrated with 95% O_2_/5% CO_2_ (pH 7.3–7.4) at room temperature. Pyramidal shaped neurons were visually identified and whole-cell recordings were established with pipettes (3–5 MΩ) containing (in mM): 130 KCl, 5 NaCl, 1 MgCl_2_, 0.2 EGTA, 10 HEPES, 2 MgATP, and 0.1 NaGTP (adjusted to pH 7.2 with KOH) to record IPSCs, or 120 mM K-gluconate, 15 KCl, 5 NaCl, 1 MgCl_2_, 0.2 EGTA, 10 HEPES, 2 MgATP, and 0.1 NaGTP (adjusted to pH 7.2 with KOH) to record evoked EPSCs. Recordings were made using an Axopatch 200A amplifier and analyzed using Clampfit and Excel. IPSCs were recorded at −70 mV in the presence of 20 µM CNQX and 50 µM APV to block excitatory AMPA/kainate and NMDA receptor mediated currents. EPSCs were recorded at −70 mV. Series resistance was 8–20 MΩ.

### Fear conditioning

#### Apparatus and data collection

Experimentally naive mice were handled daily by the experimenter for at least 6 days prior to the beginning of the experiment.

Fear conditioning experiments were performed using an automated fear-conditioning system (TSE, Bad Homburg, Germany). The apparatus consists of 4 identical conditioning test chambers (46 cm×46 cm×32 cm). Each test chamber is placed inside frames equipped with animal detection sensors and is located in a sound-attenuating box equipped with a loud speaker (for delivering white noise and acoustic stimuli), light (10 W), a ventilation fan in the side wall. The floor of the conditioning test chambers consists of a removable stainless steel foot-shock grid (bars: 4 mm diameter, distance from rod center to rod center: 8.9 mm) connected to a shock unit delivering shocks of defined duration and intensity. Conditioned stimulus (CS) and unconditioned stimulus (US) delivery were controlled by a personal computer using a program provided by TSE.

Movements of the mice were automatically registered by infrared beams spaced every 1.4 cm. Freezing behavior ( = immobility) was defined as the absence of any beam crossings for more than 1 s. For each study, freezing was automatically recorded during fear acquisition (conditioning phase) and during each subsequent session. All sessions were conducted under constant white noise (60 dB) and dim illumination (8 lux). Conditioning and testing for context-dependent freezing was performed in transparent Perspex conditioning boxes (context 1) that were cleaned with 70% ethanol between each conditioning or test session, but testing for cue-induced fear and extinction training were performed in opaque black Perspex boxes (context 2), with 2 black crosses and a black rectangle on the ceiling, that were cleaned with 1% acetic acid between each test session.

#### Single-trial fear conditioning

On day 1 the animals were individually placed into the transparent Perspex conditioning boxes (context 1) for 2 min of habituation during which time baseline freezing was recorded. Then the trial was started and 30 s later a single conditioned stimulus (CS, tone 2.8 kHz, 85 dB, 30 s) was presented that co-terminated with the unconditioned stimulus (US, 0.7 mA, 2 s, pulsed shock delivered through the grid floor, CS and US parameters from [18]). Sixty seconds after the shock the mice were returned to their home cage.

On day 2 (24 h after the conditioning), animals were tested for contextual freezing (for 3 min) in context 1. The animals were returned to their own home cage for 3 h. Then, to evaluate the cue-induced freezing, the animals were placed in the black boxes (context 2) and after 1 min (pre-CS) were presented with the tone (CS: 2.8 kHz, 85 dB, 120 s). The retention tests for context and cue were repeated 2 weeks after conditioning.

#### Multiple-trial fear conditioning and fear extinction

Fear conditioning proceeded as above in context 1 except that on day 1 the conditioning consisted of 6 pairings of the CS (tone 10 kHz, 85 dB, 30 s) co-terminating with the US (0.6 mA, 2 s, pulsed shock delivered through the grid floor) at 60 s inter-trial intervals (ITI) in context 1. Mice were returned to their home cage 60 s after the last tone/shock event.

Extinction training started 24 h after conditioning (day 2), and consisted of the presentation of 10 CS (ITI 60 s) in context 2 as above. Extinction training was then repeated in context 2 on days 3 and 4 (in total: 3×10 CS presentations). On day 2, the freezing response induced by the first 5 CS was considered as the freezing level before extinction ( = expression of cue-induced freezing, recorded in all mice). One group of animals of each genotype (wild-type and knock-out) did not receive extinction training (non-extinguished animals). These animals were returned to their home cages after 5 CS presentations on day 2 and were placed in context 2 on days 3 and 4 for 10 min without any CS or US presentation. On day 5, mice from all groups were placed in context 2 and submitted to a final retention test consisting of 10 CS presented in the absence of shock (all mice were tested).

### Intra-amygdala injections

Mice were anesthetized with ketamine/xylazine (100 mg/kg ketamine and 10 mg/kg xylazine, i.p.) and placed in a stereotaxic frame. The skull was exposed and stainless steel guide cannulae (diameter: 0.35 mm; length: 6 mm) were bilaterally implanted in the amygdala. using the following coordinates [48]: 1.5 mm caudal from Bregma, ±3.5 mm lateral from Bregma, −3.7 mm ventral from the dura. The guide cannulae were fixed to the skull with acrylic cement and 2–3 anchoring screws. The behavioral tests started following full recovery (5–6 days) of the animals from surgery. To prevent post-surgery pain, the analgesic Buprenorphin (0.01 mg/kg, i.p.) was given twice a day on the first 2 days following surgery.

On day 2 after multiple-trial fear conditioning (see below), 600 ng GRP (0.21 nmol), 3000 ng of the GRPR antagonist (D-Phe^6^,Leu-NHEt^13^,des-Met^14^)-Bombesin(6–14) (3.05 nmol) or vehicle (saline) was injected into the amygdala in awake mice 15 min before the retention test. The solutions were administered in a total volume of 0.3 µl through stainless steel cannulae injectors (diameter: 0.15 mm). Injectors were connected to a Hamilton syringe via polyethylene tubes and a microinfusion pump (CMA100, CMA, Stockholm, Sweden). The solution was slowly injected over 3 min and the injector was left in place for an additional 60 s before removal. After the infusions, mice were returned to their home cages for 10 min before starting the retention test.

#### Verification of injection sites

After behavioral testing, the animals were euthanized and 0.3 µl methylene blue was injected to mark the injection site. Brains were removed and immersion-fixed in 4% paraformaldehyde. Prior to cutting, the brains were transferred to phosphate buffered 30% sucrose for at least 12 h. Frontal sections (100 µm) were cut on a freezing microtome. Sections were mounted on gelatinized slides, counterstained with cresyl violet, dehydrated and coverslipped. The injection sites were localized and the extent of tissue lesions were examined under a light microscope. The injection sites were drawn on plates taken from a mouse brain atlas [48]. Data from animals where the injection sites were misplaced or that showed large tissue lesions were excluded from the analysis.

### Conditioned taste aversion

Mice were housed singly throughout the experiment and were pre-trained for 3 days to obtain water from a drinking tube that was present in the home cage during two 30 min periods per day. Drinking tubes were made by cutting off the tip of 15 ml Falcon® tubes to make an opening of 2–3 mm diameter. One drinking period was in the morning and one in the afternoon. Following the training period experiments were performed during the morning drinking session and only water was given in the afternoon sessions. The tubes were weighed to determine consumption. Food pellets were available *ad libitum*.

In the conditioning trial, in the morning drinking session only 0.5% saccharin solution was offered in a single tube as the stimulus designed to become the conditioned stimulus (CS). To establish a conditioned taste aversion (CTA), WT or GRPR KO mice were injected with the US, LiCl, freshly dissolved in saline (6 mEq/kg in a volume of 10 ml/kg i.p.) 30 min after the end of the saccharin drinking session. Control WT and GRPR KO mice were given saccharin to drink and injected with NaCl solution (NaCl group) or given only water to drink and injected with NaCl (saccharin-naive group).

Memory retrieval preference tests took place in the morning session 24 h after conditioning (Day 1). During the 30 min morning session, all animals were offered 2 tubes simultaneously: one filled with tap water, the other with 0.5% saccharin solution. The aversion index (AI) was calculated as follows:
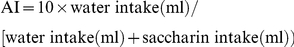
and ranged from 0 (for 100% saccharin preference) to 100 (for 100% water preference). Retrieval tests on subsequent days determined the amount of extinction of CTA.

### Attenuation of gustatory neophobia

Neophobia was determined from the difference in AI upon first exposure to a novel taste (saccharin naive) and the AI of mice that previously drank saccharin (NaCl group). The same mice used as the control groups in the CTA were assessed in this test. Attenuation of neophobia was assessed by comparing the decrease in AI seen during subsequent testing days.

### Statistical analyses

Data are presented as mean ± SEM. Statistical analyses were performed with SYSTAT (version 10 or 11 SPSS Inc.). A two-factor analysis of variance (ANOVA) with genotype and trial type as factors was used for the conditioning phase of the fear conditioning experiments after verifying that the data were normally distributed. Retention was analysed with genotype and time or cue as factors. When differences were found paired t-tests with Bonferroni corrections (24 h, 2 weeks) or Student's t-tests were applied. Extinction was compared by two-way ANOVA with genotype and extinction procedure as factors. For the pharmacological experiments, two-factor analysis of variance (ANOVA) with trial type (cue or minute) and drug treatment as factors was performed.

The conditioned taste aversion and attenuation of neophobia data were also analysed by two-factor ANOVA. If there was a significant effect of treatment or a significant group×day interaction (after Huynh-Feldt and Greenhouse-Geisser correction), individual groups were compared to the control group by means of Dunnett's multiple comparison test (two-tailed).

For the electrophysiological experiments paired t-tests were used to compare IPSC frequency before and after application of GRP. Student's t-tests were used to compare differences in LTP between WT and GRPR KO mice.

### Chemicals

Salts used to prepare solutions were from Fluka or Merck (Switzerland). GRP and the GRPR antagonist (D-Phe^6^, Leu-NHEt^13^, des-Met^14^)-Bombesin (6–14) were from Bachem AG (Switzerland). Picrotoxin was from Sigma (Switzerland). CNQX, NBQX and APV were from Tocris-Cookson (UK).

## References

[1] Buchel C, Dolan RJ (2000). Classical fear conditioning in functional neuroimaging.. Curr Opin Neurobiol.

[2] Delgado MR, Olsson A, Phelps EA (2006). Extending animal models of fear conditioning to humans.. Biol Psychol.

[3] Milad MR, Rauch SL, Pitman RK, Quirk GJ (2006). Fear extinction in rats: implications for human brain imaging and anxiety disorders.. Biol Psychol.

[4] Rogan MT, LeDoux JE (1996). Emotion: systems, cells, synaptic plasticity.. Cell.

[5] Fendt M, Fanselow MS (1999). The neuroanatomical and neurochemical basis of conditioned fear.. Neurosci Biobehav Rev.

[6] Maren S (2001). Neurobiology of Pavlovian fear conditioning.. Annu Rev Neurosci.

[7] Sah P, Faber ES, Lopez De AM, Power J (2003). The amygdaloid complex: anatomy and physiology.. Physiol Rev.

[8] Rogan MT, LeDoux JE (1995). LTP is accompanied by commensurate enhancement of auditory-evoked responses in a fear conditioning circuit.. Neuron.

[9] Rogan MT, Staubli UV, LeDoux JE (1997). Fear conditioning induces associative long-term potentiation in the amygdala.. Nature.

[10] Dityatev AE, Bolshakov VY (2005). Amygdala, long-term potentiation, and fear conditioning.. Neuroscientist.

[11] Sigurdsson T, Doyere V, Cain CK, LeDoux JE (2007). Long-term potentiation in the amygdala: a cellular mechanism of fear learning and memory.. Neuropharmacology.

[12] Etkin A, Wager TD (2007). Functional neuroimaging of anxiety: a meta-analysis of emotional processing in PTSD, social anxiety disorder, and specific phobia.. Am J Psychiatry.

[13] Phan KL, Fitzgerald DA, Nathan PJ, Tancer ME (2006). Association between amygdala hyperactivity to harsh faces and severity of social anxiety in generalized social phobia.. Biol Psychiatry.

[14] Rauch SL, Shin LM, Phelps EA (2006). Neurocircuitry models of posttraumatic stress disorder and extinction: human neuroimaging research–past, present, and future.. Biol Psychiatry.

[15] Mineka S, Oehlberg K (2008). The relevance of recent developments in classical conditioning to understanding the etiology and maintenance of anxiety disorders.. Acta Psychol (Amst).

[16] Huber D, Veinante P, Stoop R (2005). Vasopressin and oxytocin excite distinct neuronal populations in the central amygdala.. Science.

[17] Quirk GJ, Gehlert DR (2003). Inhibition of the amygdala: key to pathological states?. Ann N Y Acad Sci.

[18] Shumyatsky GP, Tsvetkov E, Malleret G, Vronskaya S, Hatton M (2002). Identification of a signaling network in lateral nucleus of amygdala important for inhibiting memory specifically related to learned fear.. Cell.

[19] De la Fuente M, Medina S, Del RM, Ferrandez MD, Hernanz A (2000). Effect of aging on the modulation of macrophage functions by neuropeptides.. Life Sci.

[20] Jeffry J, Kim S, Chen ZF (2011). Itch signaling in the nervous system.. Physiology (Bethesda).

[21] Patel O, Shulkes A, Baldwin GS (2006). Gastrin-releasing peptide and cancer.. Biochim Biophys Acta.

[22] Ehrlich I, Humeau Y, Grenier F, Ciocchi S, Herry C (2009). Amygdala inhibitory circuits and the control of fear memory.. Neuron.

[23] Ciocchi S, Herry C, Grenier F, Wolff SB, Letzkus JJ (2010). Encoding of conditioned fear in central amygdala inhibitory circuits.. Nature.

[24] Mountney C, Kent P, Anisman H, Merali Z (2006). RC-3095 administered to the basolateral nucleus of the amygdala reduces frezing to context in a fear-conditioning paradigm.. Soc Neurosci Abstr.

[25] Mountney C, Anisman H, Merali Z (2008). Effects of gastrin-releasing peptide agonist and antagonist administered to the basolateral nucleus of the amygdala on conditioned fear in the rat.. Psychopharmacology (Berl).

[26] Cao X, Mercaldo V, Li P, Wu LJ, Zhuo M (2010). Facilitation of the inhibitory transmission by gastrin-releasing peptide in the anterior cingulate cortex.. Mol Pain.

[27] Davis M, Myers KM, Chhatwal J, Ressler KJ (2006). Pharmacological treatments that facilitate extinction of fear: relevance to psychotherapy.. NeuroRx.

[28] Likhtik E, Popa D, pergis-Schoute J, Fidacaro GA, Pare D (2008). Amygdala intercalated neurons are required for expression of fear extinction.. Nature.

[29] Jungling K, Seidenbecher T, Sosulina L, Lesting J, Sangha S (2008). Neuropeptide S-mediated control of fear expression and extinction: role of intercalated GABAergic neurons in the amygdala.. Neuron.

[30] Isoardi NA, Bertotto ME, Martijena ID, Molina VA, Carrer HF (2007). Lack of feedback inhibition on rat basolateral amygdala following stress or withdrawal from sedative-hypnotic drugs.. Eur J Neurosci.

[31] Yamamoto T (2007). Brain regions responsible for the expression of conditioned taste aversion in rats.. Chem Senses.

[32] Welzl H, D'Adamo P, Lipp HP (2001). Conditioned taste aversion as a learning and memory paradigm.. Behav Brain Res.

[33] Reilly S, Bornovalova MA (2005). Conditioned taste aversion and amygdala lesions in the rat: a critical review.. Neurosci Biobehav Rev.

[34] Wahlsten D, Metten P, Phillips TJ, Boehm SL, Burkhart-Kasch S (2003). Different data from different labs: lessons from studies of gene-environment interaction.. J Neurobiol.

[35] Yamada K, Ohki-Hamazaki H, Wada K (2000). Differential effects of social isolation upon body weight, food consumption, and responsiveness to novel and social environment in bombesin receptor subtype-3 (BRS-3) deficient mice.. Physiol Behav.

[36] Merali Z, Anisman H, James JS, Kent P, Schulkin J (2008). Effects of corticosterone on corticotrophin-releasing hormone and gastrin-releasing peptide release in response to an aversive stimulus in two regions of the forebrain (central nucleus of the amygdala and prefrontal cortex).. Eur J Neurosci.

[37] Martel G, Hevi C, Wong A, Zushida K, Uchida S (2012). Murine GRPR and Stathmin Control in Opposite Directions both Cued Fear Extinction and Neural Activities of the Amygdala and Prefrontal Cortex.. PLoS One.

[38] Maren S, Quirk GJ (2004). Neuronal signalling of fear memory.. Nat Rev Neurosci.

[39] Kim JJ, Jung MW (2006). Neural circuits and mechanisms involved in Pavlovian fear conditioning: a critical review.. Neurosci Biobehav Rev.

[40] Mountney C, Sillberg V, Kent P, Anisman H, Merali Z (2006). The role of gastrin-releasing peptide on conditioned fear: differential cortical and amygdaloid responses in the rat.. Psychopharmacology (Berl).

[41] Bedard T, Mountney C, Kent P, Anisman H, Merali Z (2007). Role of gastrin-releasing peptide and neuromedin B in anxiety and fear-related behavior.. Behav Brain Res.

[42] Merali Z, Mountney C, Kent P, Anisman H (2011). Effects of intracerebral ventricular administration of gastrin-releasing peptide and its receptor antagonist RC-3095 on learned fear responses in the rat.. Behav Brain Res.

[43] Roesler R, Lessa D, Venturella R, Vianna MR, Luft T (2004). Bombesin/gastrin-releasing peptide receptors in the basolateral amygdala regulate memory consolidation.. Eur J Neurosci.

[44] Flood JF, Morley JE (1988). Effects of bombesin and gastrin-releasing peptide on memory processing.. Brain Res.

[45] Rashidy-Pour A, Razvani ME (1998). Unilateral reversible inactivations of the nucleus tractus solitarius and amygdala attenuate the effects of bombesin on memory storage.. Brain Res.

[46] Hampton LL, Ladenheim EE, Akeson M, Way JM, Weber HC (1998). Loss of bombesin-induced feeding suppression in gastrin-releasing peptide receptor-deficient mice.. Proc Natl Acad Sci U S A.

[47] Humeau Y, Shaban H, Bissiere S, Luthi A (2003). Presynaptic induction of heterosynaptic associative plasticity in the mammalian brain.. Nature.

[48] Paxinos G, Franklin KBJ (2001).

